# Calcium ionophore (A-23187) induced peritoneal eicosanoid biosynthesis: a rapid method to evaluate inhibitors of arachidonic acid metabolism *in vivo*

**DOI:** 10.1155/S0962935193000493

**Published:** 1993

**Authors:** T. S. Rao, J. L. Currie, A. F. Shaffer, P. C. Isakson

**Affiliations:** 1 Inflammatory Diseases Research Searle Research and Development c/o Monsanto Company St Louis MO 63198 USA; 2 The Salk Institute of Biotechnology/Industrial Associates, Inc. (SIBIA) 505 Coast Blvd South Suite 300 La Jolla CA 92037-4241 USA

## Abstract

The present investigation characterizes calcium ionophore (A-23187) induced peritoneal eicosanoid biosynthesis in the rat. Intraperitoneal injection of A-23187 (20 μg/rat) stimulated marked biosynthesis of 6-keto-PGF_1α_ (6-KPA), TxB_2_, LTC_4_ and LTB_4_, with no detectable changes on levels of PGE_2_. Levels of all eicosanoids decreased rapidly after a peak which was seen as early as 5 min. Enzyme markers of cellular contents of neutrophils and mononuclear cells, MPO and NAG respectively, decreased rapidly after ionophore injection; this was followed by increases after 60 min. Indomethacin, a selective cyclooxygenase inhibitor, and zileuton and ICI D-2138, two selective 5-lipoxygenase inhibitors attenuated prostaglandin and leukotriene pathways respectively. Oral administration of zileuton (20 mg/kg, p.o.) inhibited LTB_4_ biosynthesis for up to 6 h suggesting a long duration of pharmacological activity in the rats consistent with its longer half-life. The rapid onset and the magnitude of increases in levels of eicosanoids render the ionophore induced peritoneal eicosanoid biosynthesis a useful model to evaluate pharmacological profiles of inhibitors of eicosanoid pathways *in vivo*.


Mediators of Inflammation 2, 357–362 (1993)

### Calcium ionophore (A-23187) induced peritoneal eicosanoid biosynthesis: a rapid method to evaluate inhibitors of arachidonic acid metabolism *in vivo*

**T. S. Rao,[CA,*] J. L. Currie, A. F. Shaffer and P. C. Isakson**

Inflammatory Diseases Research, Searle Research and Development, c/o Monsanto Company, St Louis, MO 63198, USA

[*]*Present Address:* The Salk Institute of Biotechnology/Industrial Associates, Inc. (SIBIA), 505 Coast Blvd South, Suite 300, La Jolla, CA 92037-4241, USA

[CA] Corresponding Author

**THE present investigation characterizes calcium ionophore (A-23187) induced peritoneal eicosanoid biosynthesis in the rat. Intraperitoneal injection of A-23187 (20 μg/rat) stimulated marked biosynthesis of 6-keto-PGF$_{1\alpha}$ (6-KPA), $TxB_2$, $LTC_4$ and $LTB_4$, with no detectable changes on levels of $PGE_2$. Levels of all eicosanoids decreased rapidly after a peak which was seen as early as 5 min. Enzyme markers of cellular contents of neutrophils and mononuclear cells, MPO and NAG respectively, decreased rapidly after ionophore injection; this was followed by increases after 60 min. Indomethacin, a selective cyclooxygenase inhibitor, and zileuton and ICI D-2138, two selective 5-lipoxygenase inhibitors attenuated prostaglandin and leukotriene pathways respectively. Oral administration of zileuton (20 mg/kg, p.o.) inhibited $LTB_4$ biosynthesis for up to 6 h suggesting a long duration of pharmacological activity in the rats consistent with its longer half-life. The rapid onset and the magnitude of increases in levels of eicosanoids render the ionophore induced peritoneal eicosanoid biosynthesis a useful model to evaluate pharmacological profiles of inhibitors of eicosanoid pathways *in vivo*.**

**Key words:** ICI D-2138, Ionophore, Leukotrienes, 5-Lipoxygenase inhibitor, Zileuton

### Introduction

Activation of cyclooxygenase (CO) and/or lipoxyygenase (LO) pathways of arachidonic acid metabolism generates lipid mediators with a multitude of pharmacological activities; several of these eicosanoids have been proposed to play a major role in human inflammatory diseases such as arthritis, asthma, psoriasis, ulcerative colitis and shock.[1,2] These two pathways continue to be targeted to develop selective inhibitors with which to treat inflammatory diseases. Several animal models have been developed to evaluate biochemical efficacy and selectivity of inhibitors of arachidonic acid metabolism using either a non-immunologic or an immunologic stimulus. All of these models involve the initiation of an inflammatory response by the injection of a pharmacological agent into an anatomical site that allows measurement of indices of inflammation such as oedema, vascular permeability, cellular infiltration and generation of inflammatory mediators. In non-immunological models, materials such as carrageenin, tragacanth, zymosan, thioglycollate, calcium pyrophosphate, arachidonic acid, polymeric sponge implants and ionophores such as A-23187 have all been used to elicit inflammatory responses in diverse anatomical sites such as skin, dermis, plantar region, peritoneal and pleural cavities. In the immunological models, a specific antigen and antisera against it are injected into animals to elicit inflammation in a desired anatomical site. The indices of inflammation differ considerably in these models, perhaps reflective of their cellular and biochemical heterogeneity, and the nature of the inflammatory stimulus.[3] As a result, the utility of a given inflammation model is dictated by its characteristics such as the time course of inflammation, nature of mediators and ease of measurement of indices of inflammation.

As part of an ongoing effort to develop models suitable for rapid evaluation of selective inhibitors of CO and/or 5-LO pathways for their biochemical efficacy, selectivity and duration of action, the utility of calcium ionophore, A-23187 induced peritoneal eicosanoid biosynthesis in the rat was examined. In the present investigation, an evaluation of selective inhibitors of CO and 5-LO pathways in this model has been carried out.

### Materials and Methods

Indomethacin, A-23187, hexadecyltrimethylammonium bromide (HTAB), *o*-dianisidine 2HCl, hydrogen peroxide and Tween-80 were purchased from Sigma Chemical Co., St Louis, MO, USA. Human polymorphonuclear myeloperoxidase (MPO), bovine kidney *N*-acetyl-*β*-D-glucosaminidase (NAG), *p*-nitrophenyl-2-acetamido-*β*-D-glucopyranoside were obtained from Calbiochem (San

© 1993 Rapid Communications of Oxford Ltd

Diego, CA, USA). Selective 5-LO inhibitors, zileuton (*N*-(1-benzo[b]-thien-2-yl)ethyl)-*N*-hydroxyurea;[4] ICI D-2138 (6-(3-fluoro-5-(4-methoxy-3,4,5,6-tetrahydro-2H-pyran-4-yl)phenooxymethyl) methyl-2-quinolone);[5] were synthesized in the Chemistry Department of G. D. Searle & Co. Male Sprague–Dawley rats (140–200 g; Charles River, Portage, MD, USA) were used in all studies described here. Rats were housed in an animal vivarium with 12 h light and dark schedule. Rats were fasted overnight for oral administration of test compounds. The calcium ionophore, A-23187, was dissolved in absolute alcohol to give a working concentration of 2 mg/ml and kept at −4°C until use. On the day of the experiment, the alcoholic solution of A-23187 was diluted into phosphate buffered saline (PBS) to give a final concentration of 20 $\mu$g/ml, just prior to intraperitoneal (i.p.) injection. Indomethacin was dissolved in water with the addition of 1N NaOH (final pH 7.4). Zileuton and ICI D-2138 were administered as suspensions in 0.5% methocel and 0.025% Tween-80. All drugs were administered in a volume of 0.5 ml/rat and appropriate vehicle controls were included in each experiment. In additional experiments, protein contents in lavage samples were determined by Lowry's method and cell counts were performed using methods described in Diff-Quick Staining kit (Baxter).

*Induction of eicosanoid biosynthesis:* Groups of rats ($n$ = 6 − 8/treatment) were administered vehicle or test compounds (p.o.) and at selected times injected with A-23187 solution (20 $\mu$g/rat in 1 ml of PBS, i.p.) or PBS with 1% alcohol. Rats were killed at selected times by exposure to $CO_2$ followed by i.p. injection of 20 ml of ice-cold saline. Peritoneal contents were well mixed and 5 ml lavage samples were collected from each rat. In additional experiments, rats were injected were either saline or A-23187 180 min after an initial injection of A-23187 and rats were sacrificed 15 min after the second injection. The lavage samples were centrifuged at 32 000 × ***g*** (20 min) and supernatants used in eicosanoid assays by enzyme-linked immunosorbent (EIA) assay protocols (Cayman Chemicals, Ann Arbor, MI, USA). Levels of thromboxane $B_2$ ($TxB_2$), 6-keto-prostaglandin-$F_{1\alpha}$ (6-KPA), prostaglandin $E_2$ ($PGE_2$), leukotrienes (LT) $B_4$ and $C_4$ were measured as indices of eicosanoid biosynthesis in lavage supernatants. The pellets were used in MPO and NAG assays as described below.

*MPO and NAG assays:* Cell pellets were disrupted by sonification in 3 ml of 50 mM potassium phosphate buffer (pH 6.0), centrifuged (32 000 × ***g***, 20 min) and supernatants were discarded. Cell pellets were re-extracted into potassium phosphate buffer (50 mM, pH 6.0) with 0.5% HTAB, freeze thawed three times and centrifuged to collect supernatants which were used in MPO or NAG assays as per methods described earlier[6,7] and adapted to a 96-well plate format. Briefly, 7 $\mu$l of unknowns or human neutrophil MPO standards were added to a 96-well plate. The utility of measurements of MPO and NAG as enzyme markers for neutrophils[6] and mononuclear cells[7] respectively is well documented.

### Results

Initially, the authors attempted to define the time course of eicosanoid production and cellular changes following the injection of 20 $\mu$g of A-23187/rat. In vehicle treated rats, levels of eicosanoids remained in the range 100–500 pg/ml. Levels of $LTB_4$, $LTC_4$, $TxB_2$ and 6-KPA had rapidly increased by 5 min, followed by a rapid decline. The peak levels of eicosanoids represented 10- to 100-fold increases over the baseline levels (Fig. 1). No significant increases in levels of $PGE_2$ were seen at any time point (data not shown). Levels of MPO and NAG decreased dramatically at the first time point sampled; this was followed by increases between 60 and 300 min. In the control group, peritoneal lavage samples contained mostly neutrophils and a few mononuclear cells. Thirty min after ionophore injection, very few intact neutro-phils and mononuclear cells were seen. However, by 180 min lavage samples contained significant neutrophils and mononuclear cells (Table 1). Thus measurements of enzyme markers were corroborated by cell counts (Table 1 and Fig. 1). The changes in protein contents in lavage samples were less pronounced than changes in levels of enzyme markers (protein in $\mu$g/ml, mean ± S.E.M. ($n$ = 6); saline, 1.03 ± 0.11; ionophore (15 min), 1.62 ± 0.16; ionophore (30 min), 1.52 ± 0.19; ionophore (60 min), 2.01 ± 0.13; ionophore (120 min), 1.53 ± 0.1; and ionophore (180 min), 1.83 ± 0.23).

Interestingly, the second phase of increase in levels of MPO and NAG was not associated with any substantial eicosanoid biosynthesis. It was reasoned that a depletion of A-23187 may have contributed to a minimal eicosanoid biosynthesis at these later times. When a second injection of A-23187 was given 180 min after an initial injection of A-23187, there were substantial increases in levels of eicosanoids (Table 2) suggesting that the incoming cells are capable of synthesizing eicosanoids in response to A-23187.

For evaluation of drug effects, rats were orally administered selected doses of indomethacin or zileuton 60 min before i.p. administration of A-23187 and lavage samples were collected 15 min after A-23187 injection. Levels of 6-KPA and $LTB_4$

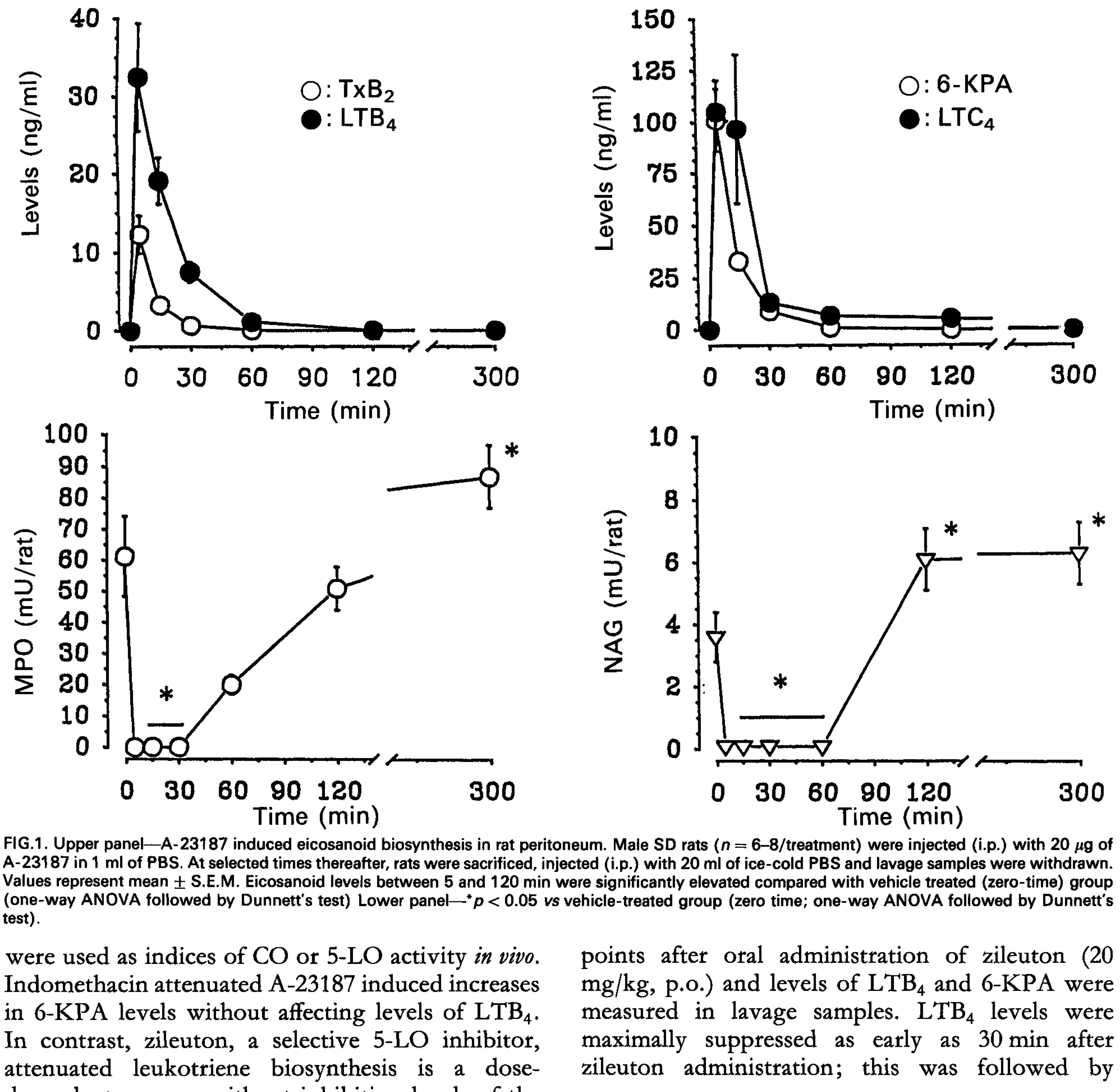

FIG.1. Upper panel—A-23187 induced eicosanoid biosynthesis in rat peritoneum. Male SD rats ($n = 6$–8/treatment) were injected (i.p.) with 20 μg of A-23187 in 1 ml of PBS. At selected times thereafter, rats were sacrificed, injected (i.p.) with 20 ml of ice-cold PBS and lavage samples were withdrawn. Values represent mean ± S.E.M. Eicosanoid levels between 5 and 120 min were significantly elevated compared with vehicle treated (zero-time) group (one-way ANOVA followed by Dunnett's test) Lower panel—*$p < 0.05$ *vs* vehicle-treated group (zero time; one-way ANOVA followed by Dunnett's test).

were used as indices of CO or 5-LO activity *in vivo*. Indomethacin attenuated A-23187 induced increases in 6-KPA levels without affecting levels of $LTB_4$. In contrast, zileuton, a selective 5-LO inhibitor, attenuated leukotriene biosynthesis is a dose-dependent manner without inhibiting levels of the CO product, 6-KPA (Fig. 2). Similar to zileuton, ICI D-2138 also selectively attenuated biosynthesis of leukotriene $B_4$. With a view to determining the utility of this model to define dynamics of inhibition of eicosanoid biosynthesis *in vivo*, rats were challenged with A-23187 for 15 min at selected time points after oral administration of zileuton (20 mg/kg, p.o.) and levels of $LTB_4$ and 6-KPA were measured in lavage samples. $LTB_4$ levels were maximally suppressed as early as 30 min after zileuton administration; this was followed by

**Table 1.** Effect of intraperitoneal injection of A-23187 on cellular composition of peritoneal lavage samples

| Treatment | Neutrophils | Macrophages | Lymphocytes |
|---|---|---|---|
| Saline | 142 ± 7 | 30 ± 3 | 29 ± 8 |
| A-23187 (30 min) | <10 | 5 ± 2 | 22 ± 7 |
| A-23187 (180 min) | 120 ± 14 | 66 ± 13 | 8 ± 3 |

500 μl of lavage samples were used in a Cytospin (600 x *g*, 10 min) followed by fixation and staining (H & E stain) as per protocols described in Diff-Quik Stain Set (Baxter Health Corp.). Two hundred cells were counted and results are presented as mean ± S.E.M. ($n = 4$–6).

**Table 2.** Effect of repeated A-23187 injection on eicosanoid biosynthesis

| Treatment | 6-Keto-$PGF_{1\alpha}$ (ng/ml)* | $LTB_4$ (ng/ml)* |
|---|---|---|
| Vehicle | 0.12 ± 0.03 | 0.01 ± 0.004 |
| A-23187 (20 μg/rat; 15 min) | 20.5 ± 6.9* | 14.9 ± 4.2* |
| A-23187 (180 min) +Vehicle (15 min) | 0.20 ± 0.02 | 0.03 ± 0.01 |
| A-23187 (180 min) +A-23187 (15 min) | 24.0 ± 4.7** | 10.1 ± 2.4** |

Male SD rats ($n = 6$/group) were injected with saline containing 1% alcohol (vehicle) or A-23187 in saline and sacrificed at times indicated above. Additional groups of mice were injected with A-23187 3 h before second injection of either vehicle or A-23187 and sacrificed 15 min later. *Values represent mean ± S.E.M.
**$p < 0.001$ *vs* vehicle or A-23187 + vehicle group.

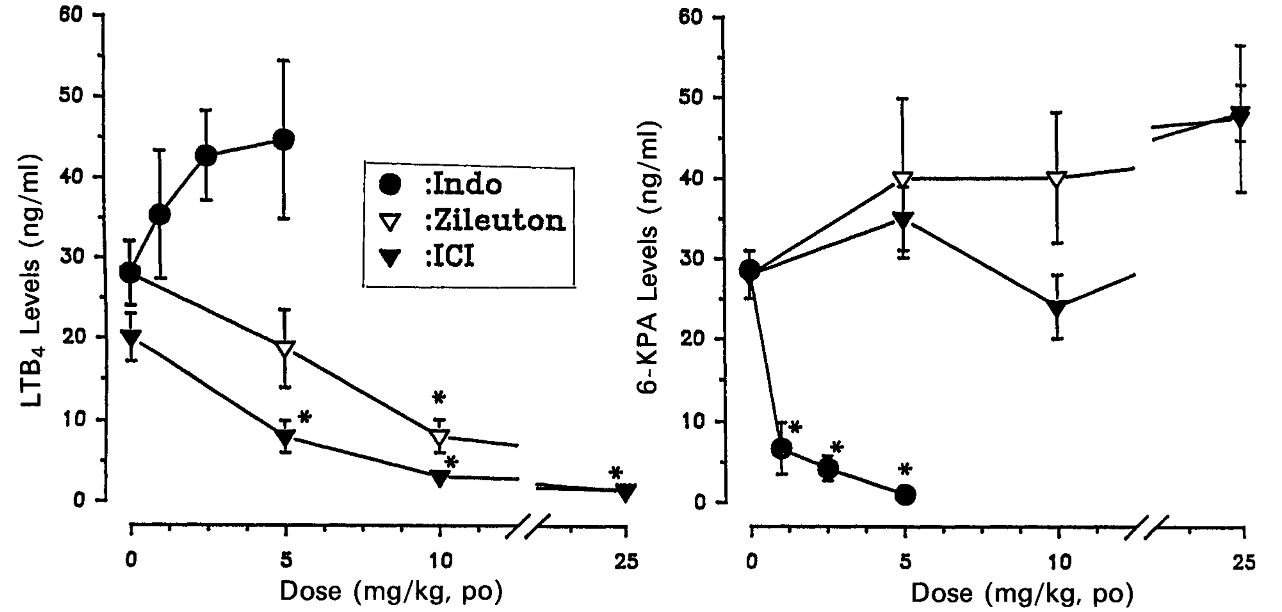

FIG.2. Effects of oral administration of indomethacin (●), ICI D-2138 (▼) or zileuton (▽) on A-23187 induced eicosanoid biosynthesis *in vivo*. Male SD rats ($n = 6$–8/treatment, fasted overnight) were administered indomethacin, zileuton or appropriate vehicle orally 60 min before i.p. injection of 20 μg of A-23187 in 1 ml of PBS. Fifteen min following A-23187 injection, 20 ml of ice-cold PBS was injected (i.p.) and lavage samples were collected. Values represent mean ± S.E.M. *$p < 0.05$ *vs* A-23187 treated group (one-way analysis of variance followed by Neuman–Keuls test).

gradual increases 6 h post administration. During the entire time course, there were no changes in levels of 6-KPA (Fig. 3). Orally administered indomethacin or zileuton did not affect ionophore induced changes in MPO despite profound inhibitory effects on mediator biosynthesis (MPO levels, mean ± S.E.M., $n = 6$–8; saline 118 ± 19; ionophore (30 min) 8.0 ± 1.4; ionophore (180 min) 147 ± 38; indomethacin (1 mg/kg), 119 ± 25; indomethacin (5 mg/kg), 139 ± 25; zileuton (5 mg/kg), 152 ± 39; and zileuton (20 mg/kg), 127 ± 19; drugs were given 30 min before ionophore injection). A similar profile was observed for NAG levels as well (data not shown).

In addition to eicosanoid biosynthesis, A-23187 injected animals exhibited considerable writhing

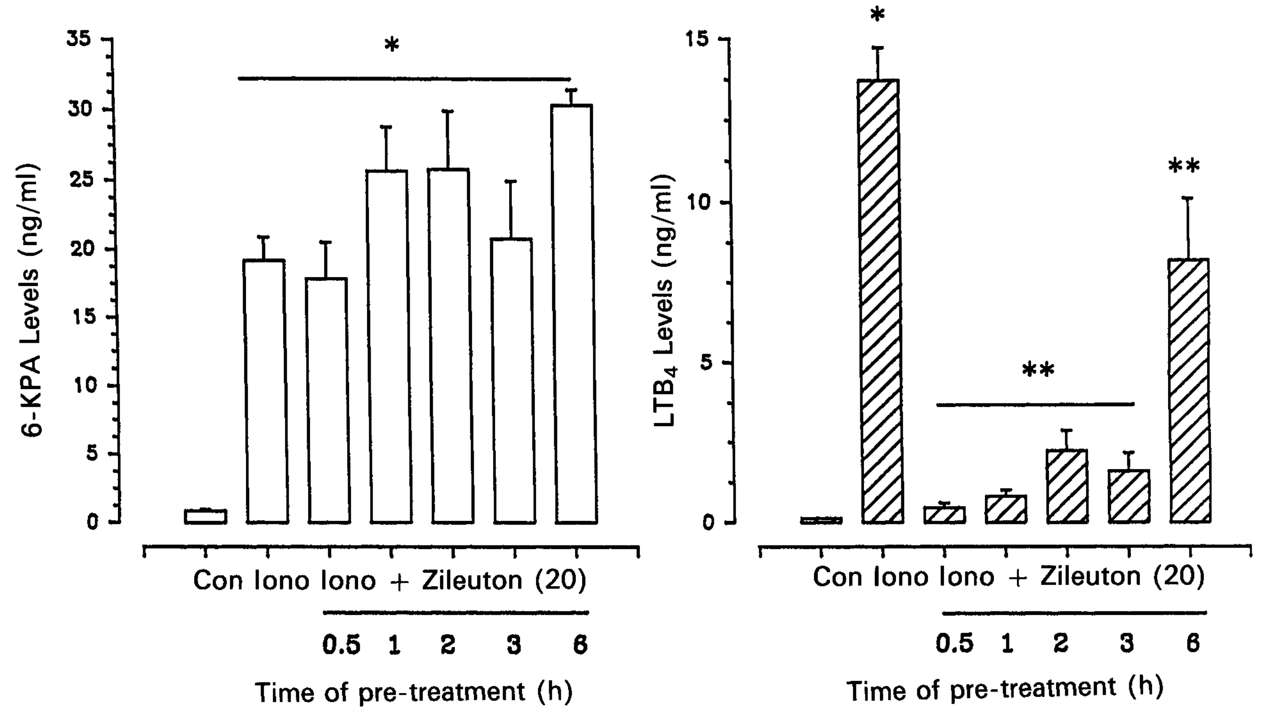

FIG.3. Time course of inhibition of eicosanoid biosynthesis following oral administration of zileuton (20 mg/kg, p.o.). Male SD rats ($n = 6$–8/treatment, fasted overnight) were administered zileuton or appropriate vehicle orally. At selected times thereafter, rats were injected with A-23187 (Iono) (20 μg in 1 ml of PBS). Fifteen min following A-23187 injection, 20 ml of ice-cold PBS was injected (i.p.) and lavage samples were collected. Values represent mean ± S.E.M. *$p < 0.05$ *vs* control (Con) group; **$p < 0.05$ *vs* A-23187 treated group (one-way analysis of variance followed by Neuman–Keuls test).

response that was attenuated by pretreatment with indomethacin, but not by zileuton or ICI D-2138 (data not shown). These observations suggest a role for eicosanoids in initiating the writhing response, consistent with earlier reports.[9]

### Discussion

Several investigators have characterized eicosanoid biosynthesis upon ionophore stimulation from a variety of cell types *in vitro*.[9–11] Subsequently the concept of ionophore induced eicosanoid biosynthesis was used in the evaluation of inhibitors of arachidonic acid metabolism in which whole blood from rodents and humans was challenged *ex vivo* with A-23187.[4,12–15] In contrast, very few studies used A-23187 *in vivo*.[9,16,17] Although *ex vivo* ionophore stimulation of whole blood is widely used for pharmacological evaluation of 5-LO/CO inhibitors, the presence of the inhibitor(s) in the blood in appropriate levels is a prerequisite for efficacy in this model. Unlike amphiphilic inhibitors, lipophilic inhibitors of arachidonate metabolism are likely to be sequestered into tissue sites and *ex vivo* eicosanoid production in blood may not reflect their *in vivo* activity under these conditions.[18] In order to evaluate effects of (lipophilic) inhibitors of arachidonic acid, ideally, arachidonic metabolism should be elicited in tissue(s) in which eicosanoid biosynthesis is minimally dependent on infiltrating cells. These issues led us to examine ionophore induced eicosanoid production in the rat peritoneal cavity as a facile means of evaluating inhibitors of eicosanoid metabolism *in vivo*. In the present investigation i.p. treatment of A-23187 resulted in rapid biosynthesis of both CO and 5-LO products, namely 6-KPA and $TxB_2$, and $LTB_4$ and $LTC_4$. In contrast to changes in mediator biosynthesis, levels of MPO and NAG, which were enzyme markers of neutrophil and mononuclear cells, decreased rapidly upon ionophore injection followed by increases at later time points, by which time eicosanoid levels tended to decrease. The initial phase of decline in levels of these two enzymes may reflect the release of intracellular granular enzymes upon activation with ionophore coincident with cellular metabolic activity and enhanced eicosanoid biosynthesis. The second phase of increase in levels of MPO and NAG may reflect influx of cells containing these enzymes into the peritoneal cavity in response to generation of chemotactic and/or chemoattractant mediators accompanied by changes in the vascular permeability within the peritoneal milieu. Differential cell counts of lavage fluids support the MPO and NAG estimates. The minimal cellular influx within the first hour of ionophore administration and a rapid onset of eicosanoid biosynthesis suggest that resident peritoneal cells are the principal source of eicosanoids in this inflammatory response.

Following the time course experiments with A-23187, effects of inhibitors of eicosanoid biosynthesis were examined with 15 min of A-23187 treatment as an end point. Although levels of eicosanoids were declining, this end point proved practical allowing ease of drug administration and collection of lavage samples. Indomethacin or zileuton and ICI D-2138 markedly attenuated prostaglandin or leukotriene pathways respectively. These results are consistent with their enzyme specificity established *in vitro* and *in vivo*.[4,5] Despite marked effects on eicosanoid biosynthesis, indomethacin or zileuton failed to attenuate A-23187 induced changes in MPO or NAG. These results suggest that products of arachidonate pathways by CO or 5-LO enzyme are minimally important in cellular infiltration in this model. These results are at variance with inhibitory effects of indomethacin and zileuton on cellular influx reported in other models[4,17] and further studies are needed to delineate these differences.

With a view to studying dynamics of inhibition of arachidonic acid metabolism, the time course of inhibition of leukotriene $B_4$ biosynthesis was examined following oral administration of zileuton. Significant attenuation of leukotriene $B_4$ biosynthesis was noted as early as 30 min and lasted beyond 3 h. These pharmocodynamic results are in agreement with a half-life of 2.3 h and $t_{max}$ (time to reach peak levels) of 15–30 min from direct measurements of zileuton levels in the rat.[4] These results clearly document the utility of this model in defining pharmocodynamic effects of inhibitors of eicosanoid metabolism.

Although immunological models of inflammation such as reverse passive Arthus reaction in the peritoneal or pleural cavities provide useful means of eliciting leukotriene biosynthesis, these responses are strongly influenced by doses of antigens and antibodies, and the requirement of high titre for the antisera.[19] In contrast, non-immunological methods such as injection of carrageenin, zymosan or arachidonic acid provide more attractive means of eliciting eicosanoid biosynthesis *in vivo*. Arachidonic acid administration elicits predominantly prostaglandin biosynthesis with smaller increases in 5-LO metabolites *in vivo*. In contract, zymosan administration results in rapid increases in prostaglandins and leukotrienes. The leukotriene production in this model is dependent on influx of phagocytic cells.[8] Agents interfering with cellular trafficking can attenuate eicosanoid biosynthesis in response to injected zymosan despite a lack of direct effects on CO and/or 5-LO enzymes. The time course of A-23187 induced peritoneal eicosanoid biosynthesis indicates a minimal role for infiltrating cells. The magnitude of increases in levels and rapid onset of biosynthesis of eicosanoids permit pharmacological manipulation by test compounds allowing determination of selectivity, potency and finally duration of action *in vivo*. These attributes render A-23187 mediated eicosanoid biosynthesis a rapid means of characterizing inhibitors of arachidonic acid metabolism *in vivo*.

### References

1. Piper PJ, Formation and actions of leukotrienes; *Physiol Rev.* 1984; **64**: 744–761.
2. Samuelsson B. Leukotrienes: mediators of immediate hypersensitivity reactions and inflammation. *Science* 1983; **220**: 568–575.
3. Chang JY, Lewis AL. Phamacological methods in control of inflammation. Alan R. Liss & Co., New York: 1989.
4. Carter GW, Young PA, Albert DH, *et al.* 5- Lipoxygenase inhibitory activity of zileuton. *J Pharmacol Exp Ther* 1991; **256**: 929–937.
5. McMillan RM, Spruce KE, Crawley GC, Walker ER, Foster SJ. Pre-clinical pharmacology of ICI D-2138, a potent orally active non-redox inhibitor of 5-lipoxygenase. *Br J Pharmacol* 1992; **107**: 1042–1047.
6. Bradley PB, Pribat DS, Christensen RO, Rothstein G. Measurement of cutaneous inflammation: estimation of neutrophil content with an enzyme marker. *J Investigative Dermatol* 1982; **78**: 206–209.
7. Bailey PJ, Sturm A, Lopez-Ramos B. A biochemical study of the cotton pellet granuloma in the rat. *Biochem Pharmacol* 1982; **31**: 1213–1218.
8. Doherty NS, Poubelle P, Bargeat P, Beaver TH, Westrich GL, Schrader NL. Intraperitoneal injection of zymosan in mice induces pain, inflammation and synthesis of peptidoleukotrienes and prostaglandin $E_2$. *Prostaglandins* 1985; **30**: 769–779.
9. Bach MK, Brashler JR. *In vivo* and *in vitro* production of a slow reacting substance in the rat upon treatment with calcium ionophores. *J Immunol* 1974; **113**: 2040–2044.
10. Bach MK, Brashler JR, Hammerstrom S, Samuelsson B. Identification of a component of rat mononuclear cell SRS as leukotriene D. *Biochem Biophys Res Commun* 1980; **93**: 211–214.
11. Jakschik BA, Kulczyki A, McDonald HH, Parker CW. Release of slow reacting substance (SRS) from rat basophilic leukemia (RBL-1) cells. *J Immunol* 1977; **119**: 618–624.
12. Gresele P, Arnout J, Coene MC, Deckmyn H, Vermylyn J. Leukotriene $B_4$ production in stimulated whole blood: comparative studies with isolated polymorphonuclear cells. *Biochem Biophys Res Commun* 1986; **137**: 334–342.
13. Ku EC, Raychaudhari A, Ghai G *et al.* Characterization of CGS8515 as a selective 5-lipoxygenase inhibitor using *in vitro* and *in vivo* models. *Biochem Biophys Acta* 1988; **959**: 332–342.
14. McMillan RM, Millest AJ, Proudman KE, Taylor KB. Evaluation of lipoxygenase inhibitors *ex vivo*. *Br J Pharmacol* 1986; **87**: 53P.
15. Sweeny FJ, Eskra JD, Carty TJ. Development of a system for evaluating 5-lipoxygenase inhibitors using human whole blood. *Prostaglandins* 1987; **28**: 73–93.
16. Hagiwara M, Mikami T, Iwamura S, Miyazawa K, Kobayashi M, Miyasaka K. Effects of TZI-41127, a novel selective 5-lipoxygenase inhibitor, on A-23187-induced pleurisy in rats. *Eur J Pharmacol* 1991; **199**: 69–75.
17. Rao TS, Currie JL, Shaffer AF, Isakson PC. Evaluation of 5-lipoxygenase inhibitors, zileuton, A-78773 and ICI D-2138 in an ionophore (A-23187)-induced pleural inflammation. *Life Sci* 1993; **53**: 147–152.
18. Bendele AM, Bensley DN, Hom JT *et al.* Anti-inflammatory activity of BF-389, a di-tertbutylphenol, in animal models of arthritis. *J Pharmacol Exp Ther* 1992; **260**: 300–305.
19. Weichman BM, Berkenkopf CA, Cullinan CA, Sturm RJ. Leukotriene $B_4$ production and pharmacologic regulation of reverse passive Arthus pleurisy: importance of antigen dose. *Agents and Actions* 1987; **21**: 351–354.

**Received 2 June 1993; accepted in revised form 5 July 1993**
